# Metal Ions Removal Using Nano Oxide Pyrolox™ Material

**DOI:** 10.1186/s11671-017-1870-x

**Published:** 2017-02-07

**Authors:** A. Gładysz-Płaska, E. Skwarek, T. M. Budnyak, D. Kołodyńska

**Affiliations:** 1 Faculty of Chemistry, Department of Inorganic Chemistry, Maria Curie Skłodowska University, M. Curie Skłodowska Sq. 2, 20-031 Lublin, Poland; 2 Faculty of Chemistry, Department of Radiochemistry and Colloid Chemistry, Maria Curie Skłodowska University, M. Curie Skłodowska Sq. 3, 20-031 Lublin, Poland; 3 Nanomaterials Department, Chuiko Institute of Surface Chemistry of National Academy of Sciences of Ukraine, 17 General Naumov St., 03164 Kyiv, Ukraine

**Keywords:** Adsorption, Toxic ions, Nanosorbent, Zeta potential

## Abstract

The paper presents the use of Pyrolox™ containing manganese nano oxides used for the removal of Cu(II), Zn(II), Cd(II), and Pb(II) as well as U(VI) ions. Their concentrations were analyzed using the atomic absorption spectrometer SpectrAA 240 FS (Varian) as well as UV-vis method. For this purpose the static kinetic and equilibrium studies were carried out using the batch technique. The effect of solution pH, shaking time, initial metal ion concentrations, sorbent dosage, and temperature was investigated. The equilibrium data were analyzed using the sorption isotherm models proposed by Freundlich, Langmuir-Freundlich, Temkin, and Dubinin-Radushkevich. The kinetic results showed that the pseudo second order kinetic model was found to correlate the experimental data well. The results indicate that adsorption of Cu(II), Zn(II), Cd(II), and Pb(II) as well as U(VI) ions is strongly dependent on pH. The value of pH 4–7 was optimal adsorption. The time to reach the equilibrium was found to be 24 h, and after this time, the sorption percentage reached about 70%. Kinetics of Cu(II), Zn(II), Cd(II), Pb(II), and U(VI) adsorption on the adsorbent can be described by the pseudo second order rate equation. Nitrogen adsorption/desorption, infrared spectroscopy (FTIR), and scanning electron microscopy (SEM) measurements for adsorbent characterization were performed. Characteristic points of the double layer determined for the studied Pyrolox™ sample in 0.001 mol/dm^3^ NaCl solution are pH_PZC_ = 4 and pH_IEP_ < 2.

## Background

Heavy metals have got wide distribution in the environment because of their multiple industrial, domestic, agricultural, medical, and technological applications. That fact is raising concerns over potential effects of heavy metals on human health and the environment [1].

Adsorption is one of the most economical, effective, and widely used methods for the removal of toxic metals from aqueous environments [2–4]. The great advantage of this method over others is the low generation of residues, easy metal recovery, and possibility of the adsorbent reuse. Plenty of approaches have been studied for the creation of ecofriendly, low-cost, and effective adsorbents for extraction of toxic contaminants such as heavy metals from aqueous solutions. For that purposes, researches studied the adsorption efficacy of natural low-cost sorbents, such clinoptilolite [5], talc [6], diatomite [7], zeolite-sewage sludge [8], peat [9], lignin [10–13], inorganic oxides [14–17], and chitosan [18–25], towards zinc, copper, cadmium, lead, vanadium, chromium, and molybdenum in waste waters that dumped to surface waters.

However, underground water generally contains iron and manganese compounds. As for manganese removal, its transformation from the soluble into insoluble form and separation of the generated oxides can be proposed. The most commonly used method is chemical oxidation combined with filtration or aeration combined with filtration by the MnO_2_ containing materials [26]. Pyrolox™ is one of such materials. This is a mineral form of manganese dioxide used in water treatment for more than 75 years. It is suitable for iron, manganese, and arsenic removal especially at pH between 5–9. However, the higher the pH the higher are the oxidation capabilities. Therefore pH 6.5 or higher is considered ideal. A pH lower than 6.5 may require extra media for contact time. Advantages of Pyrolox™ are, among others, effective reduction of iron, sulfur and manganese, long service life, low coefficient of attrition, no chemical requirement for regeneration, and periodic backwashing due to high bulk density.

This work describes the study of application of Pyrolox™ for the removal of Cu(II), Cd(II), Pb(II), and U(VI) ions. Conditions connected with the optimum pH value of the medium, interaction time, and adsorption capacity were studied. Different adsorption models were applied to describe adsorption process.

## Methods

Pyrolox™ was used for the removal of Cu(II), Cd(II), and Pb(II) as well as U(VI) ions and for description of its affinity towards the abovementioned ions. Its typical physicochemical properties are presented in Table 1.

**Table 1:** Physicochemical properties of Pyrolox^®^

	
Manufacturer	Prince Minerals, Inc.
Color	Black
Density, g/cm^3^	2.0
Bed depth, mm	Up to 600
Service flow, m/h	12
Backwash flow, m/h	60–75
Mesh size	US 8x20, US 20x40, UK 18/44
Specific gravity,	3.8
MnO_2_ content, mg/g	980
O_2_ concentration mg/dm^3^	7.28
N_2_ sorption/desorption analysis	
SEM microscan	
FTIR scan	

To characterize of the used material ASAP, FTIR and SEM methods were also applied. Additionally, the zeta potential of Pyrolox™ dispersion was determined by electrophoresis using Zetasizer Nano-ZS90 (Malvern). The electrophoretic mobility was converted to the zeta potential in millivolt using the Smoluchowski equation.

For the abovementioned purposes, the batch mode kinetic and equilibrium studies were carried out. The reaction mixtures (50 cm^3^) containing 0.5 g of adsorbent and the solution of CuCl_2_·2H_2_O, Cd(NO_3_)_2_·4H_2_O, Pb(NO_3_)_2_, UO_2_(COOCH_3_)_2_∙2H_2_O with the desired concentration of Cu(II), Cd(II), Pb(II), and U(VI) ions were prepared. In the next step, the mixtures were shaken for 360 min or 24 h and filtered. The amount of adsorbates in the adsorbent was calculated from the difference between the initial and equilibrium concentrations at time *t* using the following equation:

1 $$ {q}_t=\left({c}_0-{c}_t\right)\times \frac{V}{m} $$

The percentage adsorption of uranium from aqueous solution was computed as follows:

2 $$ S\left(\%\right)=\frac{c_0-{c}_t}{c_0}\times 100\% $$

Kinetic experiments and study of mass of adsorbent and pH influence were carried out at the initial metal concentration 112 mg/dm^3^ for Cd(II), 200 mg/dm^3^ for Pb(II), and 120 mg/dm^3^ for U(VI). The effect of pH was determined by studying the adsorption of Cu(II), Cd(II), and Pb(II) over a pH range 2–6, however, for U(VI) ions over a pH range 2–12. The pH was adjusted by the addition of HNO_3_ or NaOH solution. The pH values of the equilibrium solutions were controlled using a combined glass electrode (Sigma Chemical Co.) connected to the pH meter (CX-731 type, Elmetron Co.). The adsorption experiments were carried out using the batch method at 293, 313, and 333 K. The sorbent phase concentrations of metal ions at equilibrium, *q*_*e*_ (mg/g) and at time *t*, *q*_*t*_ (mg/g) were obtained according to the following equations:

3 $$ {q}_e=\left({c}_0-{c}_e\right)\times \frac{V}{m} $$

where *c*_0_ is the initial concentration of metal ion in the aqueous phase (mg/dm^3^), *c*_*t*_ is the concentration of metal ion in the aqueous phase at time *t* (mg/dm^3^), *c*_*e*_ is the concentration of metal ion in the aqueous phase at equilibrium (mg/dm^3^); *V* is the volume of the solution (dm^3^), *m* is the mass of the sorbent (g).

All the experimental data were the averages of triplicate determinations. The relative errors of the data were about 3%. Their concentrations were analyzed using the atomic absorption spectrometer SpectrAA 240 FS (Varian, Australia). The initial and equilibrium concentrations of U(VI) were determined spectrophotometrically using the Arsenazo III method applying the spectrometer UV-VIS V-660 (JASCO).

## Results and Discussion

### Adsorbent Characterization

The structural properties of Pyrolox™ were characterized using the nitrogen adsorption/desorption isotherms measured at 77 K. The specific surface area was determined based on the linear form of the BET equation. The pore volume (*V*_p_) was determined from that of adsorbed *N*_2_ at the pressure *p*/*p*_0_ equal to 0.98. Pore diameter (*D*_p_) was calculated according to the Eqs. 4 and 5:

4 $$ {D}_{\mathrm{p}}=\frac{\mathsf{4}{V}_{\mathrm{p}}}{{\mathrm{S}}_{\mathrm{BET}}} $$

where S_BET_ is the BET surface area and *V*_p_ is the pore volume.

Analyzing of the nitrogen adsorption/desorption isotherms and pore decomposition functions, it can be stated that the BET surface area decreases after the sorption process from 13.96 to 11.12 m^2^/g (Table 2).

**Table 2:** The BET and the Langmuir surface areas of Fe_3_O_4_ and Chitosan/Fe_3_O_4_ composites

Material	S_BET_	S_micro_	S_ext_	*V*_tot_	*V*_micro_
Pyrolox^Tm^	14 m^2^/g	2.00 m^2^/g	11.8 m^2^/g	0.022 cm^3^/g	0.009 cm^3^/g
Pyrolox^TM^–Cu(II)	11 m^2^/g	1.99 m^2^/g	10.03 m^2^/g	0.018 cm^3^/g	0.008 cm^3^/g

It was found that Pyrolox™ samples have an average pore diameter up to 7–12 nm and can be defined as mesopores. From the SEM images of Pyrolox™, it can be seen that they are of rods morphology. Analogous results were found by [27].

In the next stage, the zeta potential and the surface charge measurements were performed simultaneously. The zeta potential of Pyrolox™ dispersion was determined by electrophoresis using Zetasizer Nano-ZS90 (Malvern). The measurements were performed for suspension at concentration 100 mg/dm^3^. As a background electrolyte, NaCl solution was used at concentrations 0.1, 0.01, and 0.001 M. The electrophoretic mobility was converted to the zeta potential in millivolt using the Smoluchowski equation. To eliminate the influence of CO_2_, all potentiometric measurements were performed under nitrogen atmosphere. pH values were measured using a set of glass REF 451 and calomel pHG201-8 electrodes with the Radiometer assembly. Surface charge density was calculated from the difference of the amounts of added acid or base to obtain the same pH value of suspension as for the background electrolyte. The density of Pyrolox™ surface charge was determined using the “titr_v3” programme. Comparison of the titration curve and that of the adsorbent suspension of the same ionic strength is made to determine the surface charge density which is calculated from the dependence between the volumes of acid/base added to the suspension in order to obtain the desired pH value [28]:

5 $$ {\sigma}_0=\frac{\varDelta VcF}{S_w m} $$

where Δ*V* is the dependence between the volume of acid/base added to the suspension in order to obtain the desired pH value, *c* is the molar concentration of acid/base, *F* is the Faraday constant (9.648 × 10^4^ C/mol), *m* is the mass of the sample, *S*_*w*_ is the specific surface area of the sample.

**Fig. 1:**
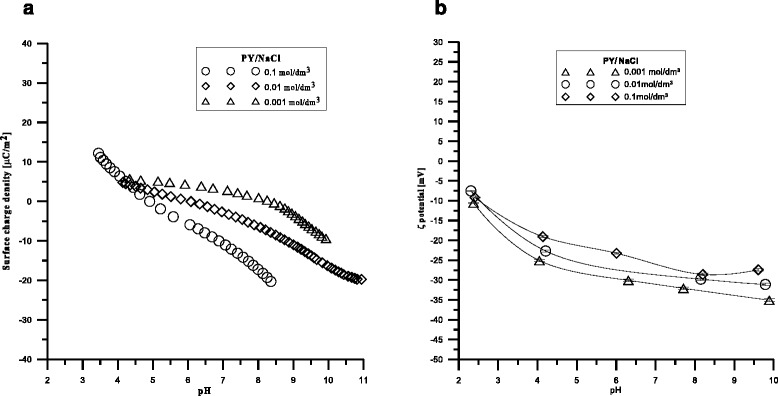
**a** Surface charge of Pyrolox™ in the aqueous solution of NaCl as a function of pH, **b** diagram of Pyrolox™ potential zeta dependence on pH value in the aqueous NaCl solutions

The point of zero charge pH_ZPC_ is defined as the point at which the surface charge equals zero. The isoelectric point pH_IEP_ is defined as the point at which the electrokinetic potential equals zero. Figure 1a presents a course of potentiometric titration of Pyrolox™ at the constant solid to liquid ratio and at three different concentrations of NaCl. The curves have a pH_PZC_ equal to 4. Figure 1b shows the electrokinetic potential as the function of pH at three different ionic strengths. It was found that at pH_IEP_ < 2 negative zeta potential was obtained.

Knowledge of the zeta potential value enables prediction of colloidal systems stability. The zeta potential allows to determine electrostatic interactions among the colloidal particles, and thus, it can be referred to the colloidal systems stability. The Pyrolox™ zeta potential allows to characterize the double electrical layer at the Pyrolox™/electrolyte solution interface. The figure of the zeta potential dependence indicates that the value of the zeta potential changes insignificantly with the pH increase for a given concentration of the electrolyte. The dependence of the zeta potential in the pH function allows to assume that pH_IEP_ has the value <2 and is lower than pH_ZPC_. With the rise of absolute value of zeta potential, generally, colloidal particles have good dispersion properties, simultaneously with the rise of electrostatic repelling, it is visible for the Pyrolox™/NaCl system.

### Kinetic and Adsorption Studies

**Fig. 2:**
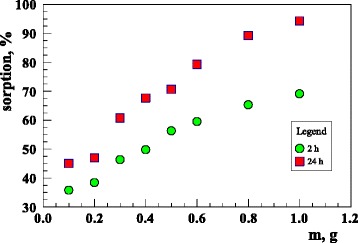
The sorbent mass influence on uranium sorption (293 K; *c*_0_ = 120 mg/dm^3^; pH = 6; *t* = 120 min or 24 h)

The dependence of U(VI) sorption on the mass of adsorbent was studied by varying the amount of the sorbent from 0.1 to 1 g, keeping other parameters (pH and contact time) constant. Figure 2 shows the U(VI) sorption capacity for the sorbent. As expected, it can be seen that the sorption capacity improves with the increasing sorbent dose in the full range up to 1 g. About 90% of U(VI) was removed when 1 g of the sorbent was added to the solution. Analogous results were obtained for Cu(II) ions.

**Fig. 3:**
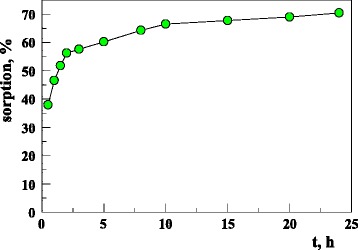
The effect of time influence on uranium sorption on the sorbent (293 K; *c*_0_ = 120 mg/dm^3^; pH = 6; 24 h)

Adsorption of U(VI) on the sorbent increased quickly at the initial contact time (Fig. 3). After 120 min from the beginning of the process, adsorption of U(VI) ions reaches 55% and becomes slower, and then achieves plateau of 70% after 24 h. The quick sorption of U(VI) on the sorbent may suggest that it was dominated by the chemical sorption rather than the physical sorption [29].

**Fig. 4:**
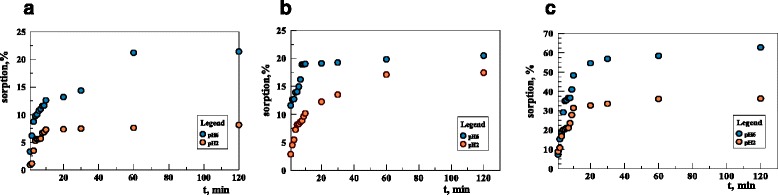
The effect of time influence on copper(II) (**a**), cadmium(II) (**b**), and lead(II) (**c**) sorption on the sorbent (293 K; *c*_0_ = 120 mg/dm^3^; pH = 6; 120 min)

As for Cu(II), Cd(II), and Pb(II) ions sorption, the efficiency of the process depends on the pH of the solution and the phase contact time of the sorbent and phase solution [30]. Figure 4a–c presents obtained results.

The equilibrium is established after the phase contact time of approximately 60 min which is much faster than in the case of U(VI); however, in this case, the process is less effective and therefore the studies were not continued. Analogous result was obtained in [31]. However, it has been shown that at pH 6, the percent of sorption of both Cu(II) and Cd(II) ions is about 20% and decreases with decreasing pH. At pH value 2.21, the percent of sorption for Cu(II) is equal to 10%. Cd(II) ions show a higher affinity than Cu(II), both at pH 2.21 and 6.22. The highest affinity was found in the case of Pb(II).

The experimental data of U(VI) adsorption on the sorbent were analyzed by two kinetic reaction models. These models were the pseudo first order and the pseudo second order [32]. Lagergren proposed a method for the analysis of adsorption which is the pseudo first order kinetic equation in the linear form:

6 $$ \log \left({q}_e-{q}_t\right)= \log {q}_e-\frac{k_1 t}{2.303} $$

where *q*_*e*_ and *q*_*t*_ are the amount of uranium ions adsorbed at equilibrium in mg/g, and at time *t* in min, respectively, and *k*_1_ is the pseudo first order rate constant (1/min).

The Lagergren first order rate constant (*k*_1_) and *q*_*e*_ determined from the model are presented in Table 3 along with the corresponding correlation coefficients.

**Table 3:** Parameters of the kinetic models for the adsorption of uranium ions on the sorbent

Model	Parameter	*T* = 293 K
Pseudo first order	*k*_1_ (1/min)	0.00368
	*q*_*e*, cal_ (mol/g)	1.65 × 10^−5^
	*R* ^2^	0.9076
Pseudo second order	*k*_2_(g/mol min)	692.2
	*q*_*e*, cal_ (mol/g)	3.59 × 10^−5^
	*R* ^2^	0.9997

The pseudo second order kinetics may be expressed as:

7 $$ \frac{t}{q_t}=\frac{1}{k^2{q}_e^2}+\frac{t}{q_e} $$

where *k*_2_ (g/mg∙min) is the second order rate constant of adsorption, *q*_*e*_ (mg/g) is the amount of uranium ions adsorbed at equilibrium, and *q*_*t*_ (mg/g) is the amount of U(VI) ions adsorbed at time *t*.

**Fig. 5:**
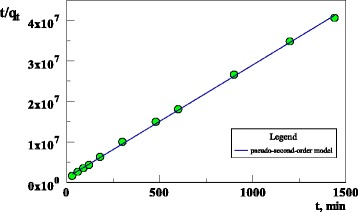
The kinetics of U(VI) adsorption on the sorbent (293 K; *c*_0_ = 120 mg/dm^3^; pH = 6; 24 h)

The equilibrium adsorption capacity (*q*_*e*_) and the second-order constants *k*_2_ can be determined experimentally from the slope and intercept of the plot *t/q*_*t*_ versus *t* (Fig. 5).

**Fig. 6:**
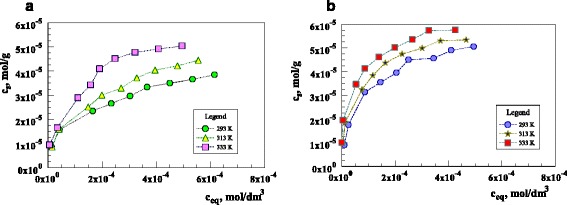
The isotherms of U(VI) adsorption on the sorbent (pHin = 6; *T* = 293, 313, 333 K; *t* = 2 h (**a**) or 24 h (**b**)) *c*_*s*_–concentration of U(VI) in the sorbent phase (mol/g); *c*_eq_–equilibrium concentration of U(VI) in the aqueous phase (mol/dm^3^)

The *k*_2_ and *q*_*e*_ determined from the model are presented in Table 2 along with the corresponding correlation coefficients. As follows, the pseudo second order model represents better the adsorption kinetics and the calculated *q*_*e*_ values agree with the experimental *q*_*e*_ values (Table 2). This suggests that the adsorption of uranium ions on the sorbent follows the pseudo second order kinetics. According to Ho and McKay [33] in the adsorption processes following the pseudo second order model, the mechanism of adsorption is mainly by chemical bonding or chemisorption [3]. The adsorption isotherms of U(VI) ions on the sorbent are shown in Fig. 6.

It can be seen that the adsorption of uranium ions on the sorbent increases with the temperature rise and proves to be the endothermic process. The U(VI) adsorption on the sorbent can be described by the Langmuir–Freundlich isotherm:

8 $$ {c}_s=\frac{a{\left({K}_{L- F}\times {c}_e\right)}^n}{\left[1+{\left({K}_{L- F}\times {c}_e\right)}^n\right]} $$

where *c*_*s*_ and *c*_*eq*_ denote the equilibrium concentrations of U(VI) in the sorbent and aqueous phases, *a* and *K*_*L–F*_ *, n* are the adsorption maximum, adsorption constant, heterogeneity parameter of the surface, respectively.

The Freundlich model was also used for analyzing the experimental sorption data:

9 $$ {c}_s={K}_F{c}_e^n $$

where *c*_*s*_ and *c*_*eq*_ denote the equilibrium concentrations of U(VI) in the sorbent and aqueous phases, *K*_*F*_ and *n* are the adsorption constant and heterogeneity parameter of the surface, respectively.

The parameter, *n*, is the heterogeneity index and varies from zero to one (the values approaching zero indicate the increasing heterogeneity and the value one indicates the homogeneous adsorbent surface). Table 4 lists the Langmuir–Freundlich and Freundlich parameters as well as the correlation coefficients (*R* ^2^).

**Table 4:** Parameters of the isotherm models for the adsorption of U(VI) ions on the sorbent

Model	Parameter	293 K 2 h	293 K 24 h	313 K 2 h	313 K 24 h	333 K 2 h	333 K 24 h
Langmuir-Freundlich	*K*_L-F_ (dm^3^/mol)	254.1	6760.8	494.3	4183.4	6006.2	7707.3
	*n*	0.459	0.721	0.542	0.607	0.748	0.768
	*q*_max_ (mol/g)	6.73 × 10^−5^	6.8 × 10^−5^	7.34 × 10^−5^	7.32 × 10^−5^	7.69 × 10^−5^	7.70 × 10^−5^
	*R* ^2^	0.9912	0.9945	0.9951	0.9958	0.9915	0.9943
Dubinin-Radushkevich	*K*_D-R_ (mol^2^/kJ^2^)	3.88 × 10^−9^	3.81 × 10^−9^	3.43 × 10^−9^	3.3 × 10^−9^	3.2 × 10^−9^	4.35 × 10^−9^
	*Q*_m_ (mol/g)	3.47 × 10^−6^	3.9 × 10^−6^	2.88 × 10^−5^	3.3 × 10^−6^	2.22 × 10^−6^	3.38 × 10^−6^
	*E* (kJ/mol)	11.35	11.61	11.76	13.38	13.56	13.72
	*R* ^2^	0.9083	0.9963	0.9929	0.9674	0.9796	0.9882
Temkin	*b*_*T*_ (J/mol)	131.6	128.3	109.7	110.5	112.8	111.8
	*K*_*T*_ (dm^3^/g)	0.970	0.956	0.947	0.952	1.002	1.005
	*R* ^2^	0.9753	0.9698	0.9898	0.9852	0.9628	0.9851
Freundlich	*K*_*F*_ (dm^3^/mol)	57.5	781.05	475.90	347.78	239.68	213.88
	*n*	0.36	0.357	0.41	0.55	0.66	0.8
	*R* ^2^	0.9642	0.9641	0.9637	0.9555	0.9622	0.9668

Examination of the data (Table 4) shows that the Langmuir–Freundlich (*R* ^2^ = 0.99) model describes the adsorption of U(VI) on the sorbent better than that of Freundlich one (*R* ^2^ = 0.96).

The adsorption capacity obtained from the Langmuir–Freundlich model is 16 mg/g (293 K), 17.4 mg/g (313 K), and 18.3 mg/g (333 K) for the sorbent.

The Temkin isotherm, in turn, contains a factor which takes into account the adsorbent–adsorbate interactions. Thus, the equation can be used to describe adsorption on heterogeneous surfaces. By neglecting the lowest and highest concentration values, the model assumes that heat of adsorption (function of temperature) of all molecules in a layer would decrease linearly rather than logarithmically with the increasing surface coverage [34]. The model in its linear form is given as:

10 $$ {c}_s=\frac{RT}{b_T}\operatorname{l} n{K}_T+\frac{RT}{b_T}\operatorname{l} n{c}_e $$

where *K*_*T*_ denotes the equilibrium binding constant (dm^3^/g), *b*_*T*_ is the constant related to the heat of adsorption (J/mol), *R* is the universal gas constant (8.314 J/(molK)), and *T* is the temperature (K). The values of the constants obtained for the Temkin isotherm are also shown in Table 4. The values of the *b*_*T*_ constant show that the heat of adsorption for the sorbent is in the range 109–131 J/mol, at different temperatures, respectively, and this fact indicates a chemical adsorption process.

The nature of adsorption can be also determined by analyzing the equilibrium data using the Dubinin–Radushkevich model (D-R) [35]. The D-R model is generally applied to express the adsorption mechanism with a Gaussian energy distribution onto a heterogeneous surface. The model has often been shown to fit both high activities of the solute and the intermediate range of concentrations. The adsorption energy is evaluated on the basis of the Dubinin–Radushkevich equation:

11 $$ {c}_s= Q m\times \exp \left(-{K}_{D- R}{\varepsilon}^2\right) $$

where *c*_*s*_ is the concentration of uranium ions in the solid phase, *Q*_*m*_ is the model constant (mol/g), *K*_D-R_ is the model constant (mol^2^/kJ^2^), and *ε* relates to the Polanyi term found from the equation:

12 $$ \varepsilon = R T\operatorname{l} n\left(1+\frac{1}{c_e}\right) $$

where *R* is the gas constant, *T* stands for the temperature, whereas *c*_*s*_ denotes the concentration of U(VI) in the equilibrium aqueous phase.

Energy of adsorption, *E*, can be calculated as:

13 $$ E=\frac{1}{{\left(2{K}_{\mathsf{D}\hbox{-} \mathsf{R}}\right)}^{0.5}} $$

The Dubinin–Radushkevich constant and mean free energy of adsorption are given in Table 4. The value of *E*_*n*_ is useful for estimating the type of sorption mechanism involved. Adsorption is physical for *E*_*n*_ < 8 kJ/mol, and chemical for 8 < *E*_*n*_ < 16 kJ/mol [36]. The values obtained for the sorbent are in the range 11–13 kJ/mol, which means that the process of adsorption of uranium ions is chemical in nature.

**Fig. 7:**
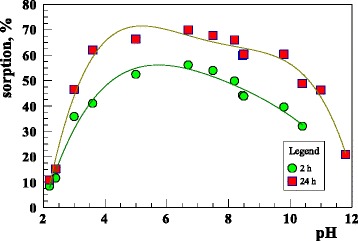
The effect of pH on U(VI) adsorption on the sorbent (293 K; *c*_0_ = 120 mg/dm^3^; pH = 6; *t* = 2 or 24 h)

pH is an important factor affecting the adsorption of uranium ions. Figure 7 shows the effect of the initial pH on the U(VI) adsorption over a range of 2–12 on the sorbent.

It can be seen that the adsorption of uranyl ions on the sorbent increases with the increasing pH values at pH < 5, reaches the highest sorption at pH 4–7 and then decreases with the increasing pH. The sorption of uranium ions on the sorbent is mainly dominated by the ion exchange or outer-sphere complexation at low pH values and by the inner-sphere complexation at high pH values [37]. The decrease of adsorption can be explained by the presence of different uranium(VI) hydrolysis products: [(UO_2_)_*p*_(OH)_*q*_]^(2*p*–*q*)+^ at different pH values and metal concentrations in the solution [36]. According to Han [35] at pH > 6 the number of dissolved anion complexes with U(VI) increases, so the adsorption decreases. The values pf pHpzc = 4 and pH_IEP_ <2 are well correlated with the results presented in Fig. 7 above pH equal 4. The surface charge density is negative, and this is the reason for such large adsorption of U(VI). Adsorption increase is observed in the pH range 2–4 which may be due to the fact that adsorption is affected by the ions of double electrical layer diffusion part which in this case is characterized by the negative zeta potential.

## Conclusions

The efficiency of the sorption process depends on the pH of solution and the phase contact time. The equilibrium is established after the phase contact time of approxiximately 60 min for Cu(II), Cd(II), and Pb(II) ions. It has been shown that at pH 6, the percent of sorption of both Cu(II) and Cd(II) ions is about 20% and decreases with the decreasing pH. The sorption process is controlled by the chemical reaction of the pseudo second order (PSO model). Cd(II) ions show a higher affinity than Cu(II), both at pH 2 and 6. In the case of the U(VI), the equilibrium is established after the phase contact time of approximately 24 h. The Langmuir-Freundlich model describes the adsorption of U(VI) on the sorbent better than the Freundlich one. Kinetic evaluation of the equilibrium data showed that the adsorption of U(VI) on the sorbent follows well the pseudo-second-order kinetic model. The adsorption energy evaluated on the basis of the Dubinin–Radushkevich equation for the sorbent are in the range 11–13 kJ/mol, which means that the process of uranium ions adsorption is chemical in nature. Adsorption of uranyl ions on the sorbent increases with the increasing pH values at pH < 5, reaches the highest sorption at pH 4–7, and then decreases with the increasing pH. The sorbent is effective for removing U(VI) from aqueous solutions. Characteristic points of the double layer determined for the studied Pyrolox™ sample in 0.001 mol/dm^3^ NaCl solution are pH_PZC_ = 4 and pH_IEP_ < 2.

## Abbreviations

D-R: Dubinin–Radushkevich isotherm model
FTIR: Fourier transform infrared spectroscopy
L-F: Langmuir–Freundlich isotherm model
*S*_BET_: Specific surface area Brunauer–Emmett–Teller
SEM: Scanning electron microscopy
